# Enhanced aging properties of HKUST-1 in hydrophobic mixed-matrix membranes for ammonia adsorption†
†Electronic supplementary information (ESI) available: Experimental procedures. See DOI: 10.1039/c5sc04368a


**DOI:** 10.1039/c5sc04368a

**Published:** 2016-01-13

**Authors:** Jared B. DeCoste, Michael S. Denny, Jr., Gregory W. Peterson, John J. Mahle, Seth M. Cohen

**Affiliations:** a Edgewood Chemical Biological Center , US Army Research, Development, and Engineering Command , 5183 Blackhawk Rd , Aberdeen Proving Ground , MD 21010 , USA . Email: jared.b.decoste2.civ@mail.mil; b Leidos, Inc. , PO Box 68, Gunpowder , MD 21010 , USA; c Department of Chemistry and Biochemistry , University of California, San Diego , La Jolla , CA 92093 , USA . Email: scohen@ucsd.edu

## Abstract

Metal–organic frameworks (MOFs) in their free powder form have exhibited superior capacities for many gases when compared to other materials, due to their tailorable functionality and high surface areas. Specifically, the MOF HKUST-1 binds small Lewis bases, such as ammonia, with its coordinatively unsaturated copper sites. We describe here the use of HKUST-1 in mixed-matrix membranes (MMMs) prepared from polyvinylidene difluoride (PVDF) for the removal of ammonia gas. These MMMs exhibit ammonia capacities similar to their hypothetical capacities based on the weight percent of HKUST-1 in each MMM. HKUST-1 in its powder form is unstable toward humid conditions; however, upon exposure to humid environments for prolonged periods of time, the HKUST-1 MMMs exhibit outstanding structural stability, and maintain their ammonia capacity. Overall, this study has achieved all of the critical and combined elements for real-world applications of MOFs: high MOF loadings, fully accessible MOF surfaces, enhanced MOF stabilization, recyclability, mechanical stability, and processability. This study is a critical step in advancing MOFs to a stable, usable, and enabling technology.

## Abstract 2

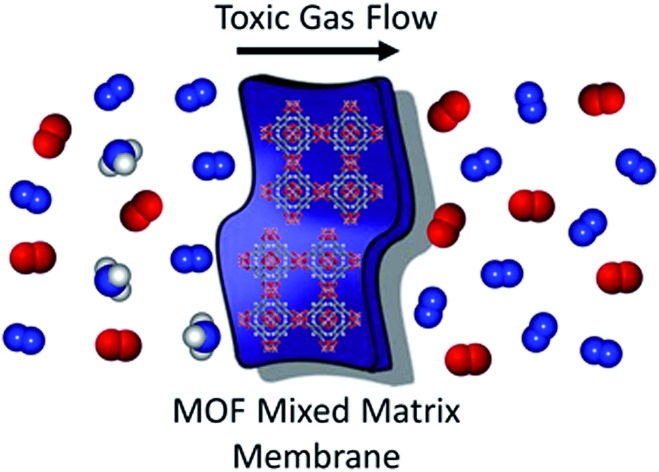
The metal-organic framework (MOF) HKUST-1 incorporated into a mixed-matrix membrane (MMM) exhibits enhanced water stability while maintaining gas removal capabilities commensurate with those of the free powder form.

## Introduction

With an annual production over 200 million tons, ammonia is one of the most widely manufactured chemicals in the world.^1^ Ammonia has been identified as a chemical that frequently creates a high risk for accidents such as spills at manufacturing facilities or explosions at fertilizer plants.^2^ Furthermore, the availability and toxicity of ammonia make it a potential chemical for insurgents to utilize in asymmetric warfare. In the 1990s, Serbian forces targeted chemical plants during the war in Croatia as a method of attack toward civilians by causing ammonia release into the environment.^3^ For these reasons, the development of engineered materials that can remove large amounts of ammonia for air purification applications is paramount.

Metal–organic frameworks (MOFs) are porous materials comprised of inorganic metal nodes, known as secondary building units (SBUs), linked together by polydentate organic ligands.^4^,^5^ The various combinations of SBUs and organic linkers allow for tuning of the physical and chemical properties. Many MOFs are microporous, making them ideal for gas storage,^6^–^9^ gas separations,^10^,^11^ molecular sensing,^12^,^13^ toxic chemical adsorption,^14^,^15^ and catalysis.^16^,^17^ While MOFs have been examined extensively in their primitive powder form, far fewer studies have been conducted on their properties in engineered forms or as part of a matrix.^18^–^22^

To incorporate MOFs into materials for applications in textiles, filters, or sensors, engineered forms, such as pressed pellets, particles with binders, or films, must be fabricated. Studies of pure-MOF membranes have been reported, but typically only small area samples can be achieved, and delamination typically proves difficult.^23^ Mixed-matrix membranes (MMMs) of MOFs have been reported primarily for the study of gas separations. MMMs have the potential to enhance the utility of MOFs by allowing for the facile fabrication of supported or freestanding films with variable material composition that exhibit mechanical and material properties beyond that of single crystals or free flowing powders. Recently, the use of polyvinylidene difluoride (PVDF) to prepare MMMs for a wide variety of MOFs was described.^24^ PVDF-based MMMs of MOFs exhibited good mechanical properties, while allowing for high weight percent loading of MOFs. Furthermore, the crystallinity, surface area, and chemical reactivity of the MOFs was largely unperturbed in these MMMs.

**Fig. 1:**
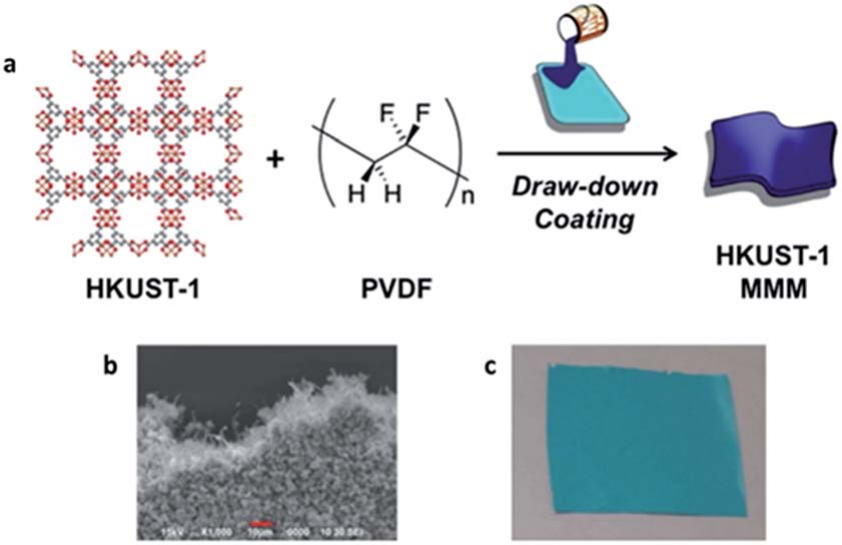
Schematic depicting the fabrication of mixed-matrix membranes of HKUST-1. (a) 3-dimensional structure of HKUST-1, chemical structure of polyvinylidene difluoride, and schematic representing the MMM fabrication process, (b) SEM image of 67-HKUST-1 MMM and (c) picture of 67-HKUST-1 MMM.

HKUST-1 (aka. Cu–BTC, Cu_3_(BTC)_2_, MOF-199, HKUST-1 = Hong Kong University of Science and Technology) is a copper based MOF, in which paddlewheel Cu-dimers are linked together by benzene 1,3,5-tricarboxylate to form a 3-dimensional pore structure, as seen in Fig. 1a.^25^ HKUST-1 has been shown to be superior to other MOFs for the adsorption of basic gases such as ammonia, due to coordinatively unsaturated Cu-sites.^26^,^27^ Recently, HKUST-1 was incorporated into biological chitin fibers at loadings up to 55% (w/w), while maintaining approximately 75% of its ammonia capacity (based on HKUST-1 content).^28^

One of the major shortcomings of HKUST-1 is its instability toward liquid water and high humidity conditions.^29^–^31^ Upon exposure to 90% RH at 25 °C, HKUST-1 exhibits a substantial loss of its porosity and ammonia uptake capacity, with a transformation of the crystal structure.^31^ It has been shown that the chemical stability of HKUST-1 can be enhanced *via* plasma enhanced chemical vapour deposition of perfluoroalkanes into the MOF structure.^32^,^33^ The hydrophobic nature of fluoroalkanes prevents water molecules from clustering in the pores and subsequently breaking the Cu–carboxylate linkages. Despite the success of this approach, it does not address the myriad of other issues required to make a suitable gas capture device from MOFs, including retaining fully accessible MOF surfaces, facile recyclability, mechanical stability, and processability. Here we address all of these issues with a single solution. We explore the use of a hydrophobic polymer (PVDF) to prepare HKUST-1 MMMs of various composition for ammonia removal, as well as the effect of humidity on the performance of the MOF in the MMM. We find that the ammonia removal performance of HKUST-1 in these MMMs is unprecedented, with greatly improved stabilization of the MOF toward both humidity and ammonia, when compared to HKUST-1 powder.^31^

## Results and discussion

### Mixed-matrix membrane preparation

The preparation and initial characterization of PVDF MMMs with a variety of MOFs was recently reported.^24^ Fabrication of these MMMs, described in depth in the ESI,† involves an ink comprised of a solution of PVDF polymer and HKUST-1 that is applied to a substrate followed by solvent evaporation to form a film that can be delaminated to give a freestanding MMM (Fig. 1). The samples throughout this manuscript are named [wt%]-HKUST-1 MMM, where [wt%] = 30, 50, or 67. For the MMMs, as the HKUST-1 content increases, so does the intensity of the HKUST-1 peaks in the PXRD spectra (Fig. S1†). Likewise, the FTIR spectra (Fig. S2†) show that as the PVDF/HKUST-1 content varies for each sample, the corresponding infrared bands for PVDF and HKUST-1 vary in the same manner.

### Ammonia adsorption

Ammonia uptake capacities were measured for each material using dynamic microbreakthrough tests at a concentration of 2000 mg m^–3^ (see ESI†). The ammonia capacities, as determined from the breakthrough curves (Fig. S3†), for the HKUST-1 powder and HKUST-1 MMMs are shown in Table 1. HKUST-1 exhibited an ammonia capacity of 7.4 mol kg^–1^, which is commensurate with a loading of approximately 1.5 NH_3_ molecules per Cu atom. It has been shown that even at low pressures each Cu atom of HKUST-1 ligates one NH_3_ molecule.^27^ The additional ammonia sorption is likely due to hydrogen bonding with the chemisorbed NH_3_ molecules in the MOF pore. The ammonia capacity of each MMM varies proportionally with HKUST-1 content. Interestingly, as the HKUST-1 content increases in the MMM, the experimental ammonia capacity agrees better with the hypothetical capacity, as determined from the weight percent of HKUST-1 in each MMM. The good agreement between the experimental and hypothetical capacities, especially for 50-HKUST-1 and 67-HKUST-1 MMM, is strong evidence that the HKUST-1 crystals even within the interior of the MMM are largely accessible to the contaminated airstream.

**Table 1:** Experimental and hypothetical ammonia loadings for HKUST-1 MMMs

Sample	Ammonia loading (mol kg^–1^)	Hypothetical ammonia loading (mol kg^–1^)
PVDF polymer	0.1	N/A
30-HKUST-1 MMM	1.3	2.2
50-HKUST-1 MMM	3.2	3.7
67-HKUST-1 MMM	4.9	5.0
HKUST-1 powder	7.4	N/A

The pre- and post-ammonia exposed HKUST-1 MMMs, HKUST-1 powder, and PVDF were analyzed using PXRD (Fig. S1 and S4†) and FTIR (Fig. S2 and S5†). The HKUST-1 powder exhibited a change in the PXRD pattern upon exposure to ammonia, with new reflections at 2*θ* = 18.1, 25.3, and 27.0°, indicative of a substantial phase change and the loss of the HKUST-1 structure. In contrast, the PXRD patterns of each HKUST-1 MMM showed minimal change, indicating that HKUST-1 maintains its crystallinity better in the MMMs upon exposure to ammonia, when compared to the HKUST-1 powder.

HKUST-1 powder exposed to ammonia also showed a loss of the FTIR band at 1646 cm^–1^, which is indicative of Cu–carboxylate bonding.^31^ These characteristics are consistent with ammonia degrading the MOF structure, as seen in earlier reports.^26^ The appearance of the bands at 1610 cm^–1^ in the FTIR spectrum upon the exposure of HKUST-1 to ammonia is characteristic N–H bending mode, indicative of the presence of ammonia.^34^ The FTIR spectra of the HKUST-1 MMMs upon exposure to ammonia show that the Cu–carboxylate band is retained, which is consistent with the PXRD data confirming the retention of the HKUST-1 crystal structure. Furthermore, the HKUST-1 MMMs displayed an N–H bend at 1610 cm^–1^ indicative of ammonia binding. The presence of Cu–carboxylate and N–H modes shows that even upon ammonia adsorption HKUST-1 can be stable when confined in a PVDF MMM. Taken together, the microbreakthrough, PXRD, and FTIR data show that ammonia sorption in HKUST-1 powder induces rapid degradation, but that incorporation of HKUST-1 into a MMM stabilizes the MOF, without loss of ammonia sorption capacity.

Typically, engineered forms of sorbent materials have decreased activity toward an analyte of interest due to the blocking of pores and/or active sites. However, that is not observed here, likely due to the vast majority of active sites being located within the micropores of the MOF, instead of on the outer surface as is typically the case with metal/metal oxide nanoparticles. Even though many of the outer MOF pores may be in contact with the polymer binder, many of these pores must still be accessible such that ammonia can diffuse into the MOF crystallites, as evidenced by the high capacities observed for HKUST-1 MMMs. Furthermore, based on the sorption capacity observed here, it is improbable that the PVDF polymer penetrates into the inner pores of the MOF, impeding the diffusion of adsorbates within the MOF.

### Effect of water on HKUST-1 MMMs

It was previously found that HKUST-1 in its powder form degrades upon exposure to humid conditions over the course of weeks.^31^ The most aggressive aging condition was found to be 90% RH at 25 °C, corresponding to an absolute humidity of 20.5 g m^–3^, where HKUST-1 has a water uptake of 32 mol kg^–1^ or 38 wt%. We used these same conditions in this study to examine the moisture stability of the HKUST-1 MMMs.

**Fig. 2:**
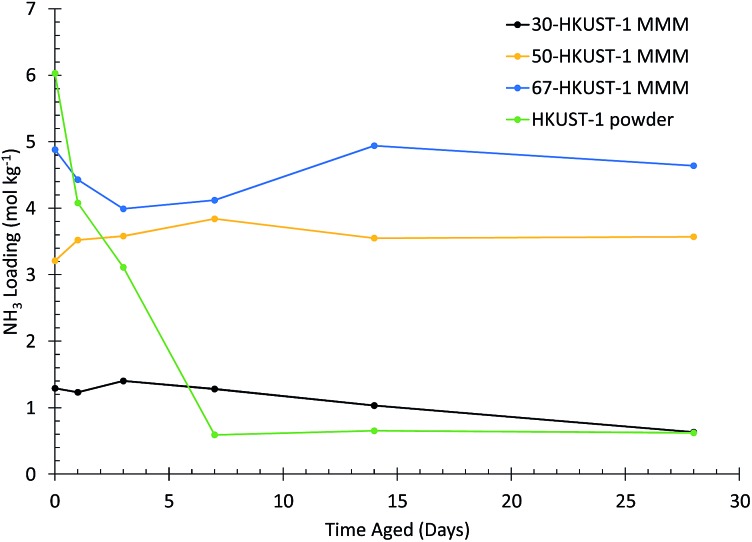
Ammonia loading for HKUST-1 (powder) and HKUST-1 MMMs. Different HKUST-1 loadings after aging at 90% RH and 25 °C for various amounts of time are shown.

The ammonia loading for each HKUST-1 MMM, compared to the HKUST-1 powder,^31^ after aging for various times is shown in Fig. 2 (as determined from the microbreakthrough experiments in Fig. S6–S8†). For the HKUST-1 powder, ∼90% of the ammonia capacity is lost in the first 7 days, without much further change over the full 28 days of the experiment. In contrast, for the 50-HKUST-1 and 67-HKUST-1 MMMs the ammonia capacity over the full 28 days of aging varies less than 20%. 30-HKUST-1 MMM shows ∼20% loss in ammonia capacity after aging for 14 days, and ∼50% after aging for 28 days. Although 30-HKUST-1 MMM loses more ammonia capacity than higher loading MMMs, the relative loss in capacity of 30-HKUST-1 is still substantially less than the HKUST-1 powder.

Upon examination of the PXRD patterns (Fig. 4) of the HKUST-1 MMMs, it becomes more evident that the structures of 50-HKUST-1 and 67-HKUST-1 MMMs withstand the harsh aging conditions that HKUST-1 powder cannot. In general, the PXRD pattern remains unchanged over the 28 days studied for these materials. Furthermore, as expected from the loss in ammonia capacity of the 30-HKUST-1 MMM as it is aged, a degradation in the HKUST-1 crystal structure is observed. Interestingly, there is no formation of the secondary crystal structure in 30-HKUST-1 MMM that is seen upon degradation of the HKUST-1 powder, evidenced by reflections at 2*θ* ≈ 7.9, 9.2, 12.1, and 14.3°. It should also be noted that the HKUST-1 PXRD reflections are observed in 30-HKUST-1 MMM after 28 days of aging, but they are decreased in intensity.

**Fig. 3:**
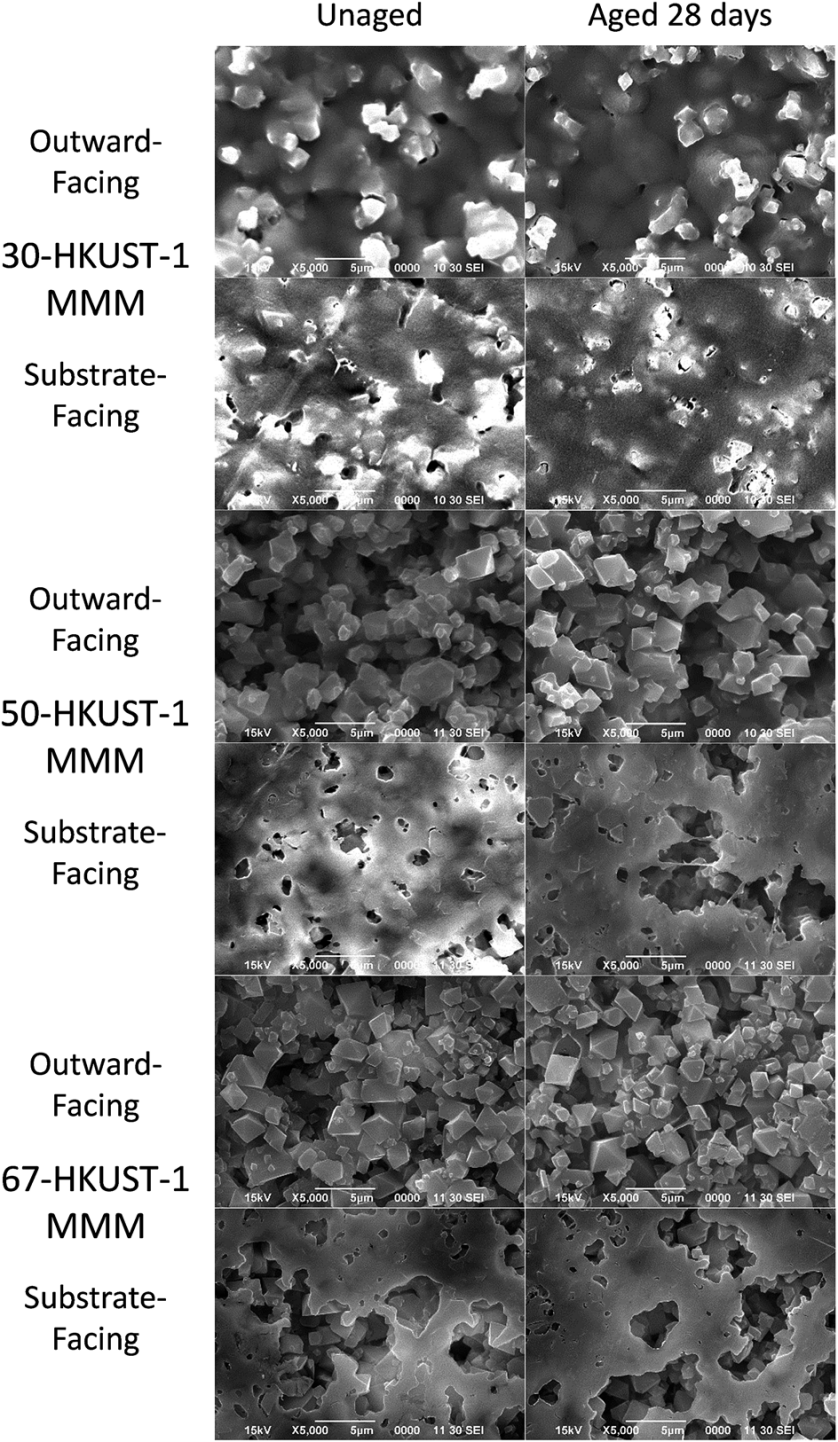
SEM images of 30-HKUST-1, 50-HKUST-1, and 67-HKUST-1 shown from the outward-facing (MOF dominant) side and substrate-facing (polymer dominant) side before (left) and after (right) aging at 90% RH at 25 °C for 28 days.

**Fig. 4:**
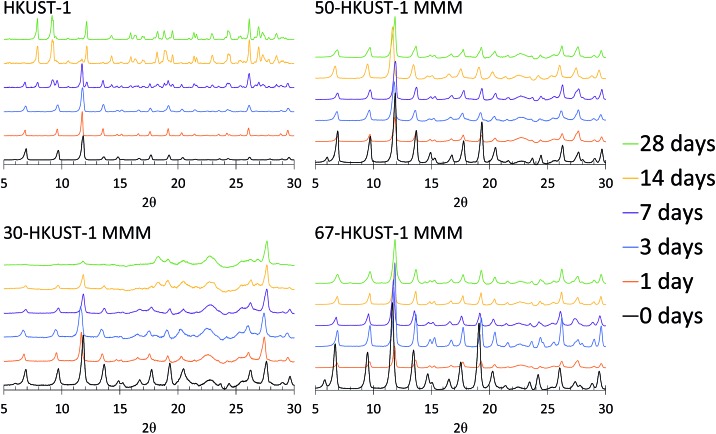
PXRD patterns of 30-HKUST-1, 50-HKUST-1, and 67-HKUST-1 MMMs, compared to HKUST-1,^31^ aged for 0, 1, 3, 7, 14, and 28 days at 90% RH at 25 °C.

In previous work, we identified the breakdown of the HKUST-1 structure by water to proceed mechanistically through the breaking of the Cu–carboxylate bond to form a carboxylic acid, seen through FTIR bands at 1708 and 1243 cm^–1^.^31^ Over the course of 28 days we did not observe the appearance of these FTIR bands for any of the MMMs (Fig. S9–S12†). Interestingly, FTIR bands at 1620 and 1540 cm^–1^, which represent physisorbed water, were observed for 30-HKUST-1 MMM, which was also the only MMM that showed any signs of degradation. These same bands were observed in the powder form of HKUST-1 at the early stages of MOF degradation (days 1–3), with a modest decrease in the ammonia uptake, but no noticeable change in the crystal structure was observed. Conversely, these FTIR bands are not observed in 30-HKUST-1 MMM until day 14, nor are they observed in the 50-HKUST-1 and 67-HKUST-1 MMMs over the 28 day study. More importantly none of the HKUST-1 MMMs show the appearance of the carboxylic acid modes typically observed upon breakdown of HKUST-1.

Physically no change was observed in the color or appearance of the 50-HKUST-1 and 67-HKUST-1 MMMs during the aging process; however, 30-HKUST-1 MMM showed a fading of the characteristic blue color from what appeared to be visible degradation of the material (Fig. S13†). The preparation of the MMMs results in the side against the substrate having greater polymer content (referred to as substrate-facing side), while the other side is richer in MOF crystals dispersed throughout the PVDF (referred to as outward-facing side), which is more representative of the bulk MMM. SEM images (Fig. 3 and S14†) further show the physical state of the 50-HKUST-1 and 67-HKUST-1 MMMs remain essentially unchanged over the 28 days of aging.

The different sides of the MMM have varying degrees of hydrophobicity, as do the MMMs with various amounts of HKUST-1, as determined by contact angle measurements (Table 2). Two distinctive trends were observed: (1) the contact angle increases as the HKUST-1 content of the MMM increases, and (2) the outward-facing side of the MMM has a higher contact angle than the substrate-facing side of the MMM. The water contact angle of a material is driven by not only the chemical make-up of the material, but also by the surface roughness.^35^,^36^ The air trapped in the space between the MOF particles is an important contributor to the increased hydrophobicity as the water contact angle of air is considered to be 180°.^36^ We propose that the increased surface roughness from the individual MOF crystals is the primary contributor to the increased contact angle of the outward facing-side (more MOF-like) of the MMMs verse the substrate-facing side (more PVDF-like). As expected, due to increased surface roughness, the HKUST-1 MMMs with a higher percentage of HKUST-1 exhibit an increased contact angle. Of particular significance, the water contact angle of an HKUST-1 pellet has been reported to be 59°,^33^ much lower than that observed for the any of the MMMs.

**Table 2:** Contact angles for each HKUST-1 MMM measured on the outward facing (MOF dominant) and substrate facing (polymer dominant) side

Sample	Side	Water contact angle
PVDF polymer	N/A	82° ± 3
30-HKUST-1 MMM	Outward	84° ± 1
	Substrate	80° ± 1
50-HKUST-1 MMM	Outward	101° ± 1
	Substrate	83° ± 2
67-HKUST-1 MMM	Outward	110° ± 1
	Substrate	107° ± 0

The increased hydrophobicity of the MMMs compared to HKUST-1 powder causes a significant decrease in the total water uptake as observed in the water isotherms performed at 25 °C (Fig. S15 and S16†). When corrected for MOF content, the water loading is similar for each of the MMMs. It has been shown elsewhere that hydrolysis of HKUST-1 requires the clustering of water molecules near the Cu–carboxylate bonds, in order for hydrolysis to occur.^30^ The decreased water uptake of the MMMs causes there to be less water per SBU, significantly decreasing the potential for degradation of the MOF *via* hydrolysis.

The PXRD and FTIR data, along with the ammonia capacities of the HKUST-1 MMMs, clearly show that the 50-HKUST-1 and 67-HKUST-1 are quite stable to humid environments over the period studied here. As seen previously, the presence of hydrophobic fluoroalkanes enhance the water stability of HKUST-1.^32^,^33^ However, in humid environments 30-HKUST-1 begins to lose its crystallinity and consequently its ammonia adsorption capacity over time, due to the early stages of MOF degradation, which can be observed through the sorption of water in the FTIR spectra, and loss of the HKUST-1 crystal structure in the PXRD spectra. 30-HKUST-1 MMM contains the highest ratio of the PVDF polymer, which in the case of the MMM acts as a binder and supplies hydrophobic CF_2_ groups. Intuitively, one might hypothesize that the 30-HKUST-1 MMM would withstand the humid environment that the materials were subject to better than the MMMs with a higher HKUST-1 content; however, this was not the case. It was observed that the 30-HKUST-1 MMM sample had much lower contact angles (similar to that of PVDF) than the other MMMs, likely due to the increased surface roughness of the 50-HKUST-1 and 67-HKUST-1 MMMs. In the SEM images of the higher HKUST-1 content MMMs, it can be observed that the MOF crystals dominate the surface of the MMM, which is not the case for 30-HKUST-1 MMM. In turn, the flattened surface of 30-HKUST-1 MMM does not repel water droplets as well as the other MMMs, and over the course of 28 days may attract more water into the MOF structure, promoting eventual degradation of the MOF, even though this was not observed on the timescale of the water isotherm. Nevertheless, the degree of degradation for the MMM is much less than is observed for the pure HKUST-1 powder. In fact, after 7, 14, and 28 days of aging, the 30-HKUST-1 MMM even has a higher ammonia capacity than the HKUST-1 powder, despite 30-HKUST-1 MMM only having 30% of the material being the active HKUST-1 MOF.

## Conclusions

HKUST-1 MMMs are strong candidates for use in gas filtration applications, due not only to the high ammonia capacities of the engineered form of the MOF, but due to the increased water and ammonia stability of the MOF over the primitive powder form. Furthermore, the MMMs can easily be shaped or molded into useful forms for a given application. PVDF acts as an effective binder for HKUST-1, while still allowing the pores to be permeable and accessible to adsorbates such as ammonia, giving ammonia capacities that are scalable to the HKUST-1 content of the MMM.

Remarkably, no degradation was observed over the course of 28 days for the 50-HKUST-1 and 67-HKUST-1 MMMs upon exposure to 90% RH at 25 °C. Furthermore, the ammonia capacity for these samples was relatively constant over the period studied. The increased water stability of HKUST-1 in the MMM is due to the increased chemical hydrophobicity stemming from the fluorocarbon groups of the PVDF polymer and the surface roughness created by the MOF crystals bound in the matrix, making these materials resistant towards both water vapor and liquid water. Overall the findings here strongly support the use of MOF MMMs as a means to enhance the utility, processability, stability, and performance of MOFs in gas sorption applications in a comprehensive manner that has not been demonstrated before.

## Supplementary Material

Supplementary information
