# Supplementary material for: Enhanced aging properties of HKUST-1 in hydrophobic mixed-matrix membranes for ammonia adsorption
Source: Chem Sci. 2016 Jan 13;7(4):2711–6. doi: 10.1039/c5sc04368a (PMC5477018; doi:10.1039/c5sc04368a)
Supplement: Supplementary file 1 [file SC-007-C5SC04368A-s001.pdf]

Electronic Supplementary Material (ESI) for Chemical Science.
This journal is © The Royal Society of Chemistry 2016

Enhanced Aging Properties of HKUST-1 in Hydrophobic Mixed-Matrix Membranes for Ammonia Adsorption

Jared B. DeCoste,[a,b] Michael. S. Denny, Jr.,[c] Gregory W. Peterson,[a] John J. Mahle,[a] and Seth M. Cohen[c]**

[a]Edgewood Chemical Biological Center, US Army Research, Development, and Engineering Command, 5183 Blackhawk Rd., Aberdeen Proving Ground, MD 21010, United States

[b] Leidos, Inc., PO Box 68, Gunpowder, MD 21010, United States

[c] Department of Chemistry and Biochemistry, University of California, San Diego, La Jolla, CA 92093, United States

Supporting Information

Experimental Procedures

Mixed Matrix Membrane Fabrication

First, HKUST-1 was synthesized by dissolving copper(II) nitrate hemipentahydrate ($Cu(NO_3)2\cdot2.5H2O$) (1.22 g, 5.24 mmol) and 1,3,5-benzenetricarboxylic acid (0.58 g, 2.76 mmol) in 5 mL DMSO. This solution was then added dropwise at room temperature to 250 mL of MeOH with magnetic stirring over the course of 15 min. Stirring was continued for 15 min after complete addition. The particles were collected by centrifugation, washed with 3×10 mL portions of MeOH, and dried under vacuum at room temperature.[1]

The resulting dry HKUST-1 powder was dispersed in 5 mL acetone with sonication for 30 min. A PVDF solution (7.5 wt% in DMF) was then added to the MOF suspension giving the desired HKUST-1: PVDF ratio, and sonicated for 30 min. The acetone was removed via rotary evaporation, resulting in a MOF 'ink'. The ink was cast into films on Al substrates by drawdown coating with a glass rod (spacer thickness 300-400 µm). The resulting film was heated at 70°C for 1 h to remove solvent. The resulting membranes were delaminated via immersion in solvent (acetone, MeOH). The free standing films were air dried.[2]

Microbreakthrough experiments

A miniaturized breakthrough apparatus was used to evaluate milligram-scale quantities of samples for the adsorption of ammonia.[3,4] Samples measuring approximately 1 sq. inch were rolled up and inserted into a nominal 4 mm i.d. fritted glass tube that was subsequently loaded into a water bath for isothermal testing at 20 °C. Prior to testing, each material was regenerated for 1 h at 100 °C under flowing dry air to remove any physisorbed water, then weighed. A ballast

with a predetermined quantity of challenge gas was then mixed with a stream of dry (-40°C dew point) air at a rate necessary to achieve a challenge concentration of 2,000 mg m^{-3}. The contaminated air stream was then sent through the fritted glass tube at a flow rate of 20 mL min^{-1}. The effluent stream was sent through a photoionization detector to monitor the ammonia concentration.

The data is reported and plotted as normalized time (the time divided by the mass of the sample used) verse the signal at a given time divided by the signal at saturation (C/C_0). The corresponding breakthrough curve was integrated to determine the dynamic capacity to saturation.[3,4] First, a concentration time number, Ct, is defined as:

$$Ct = t_*C \quad (1)$$

Where t is the time and C is the concentration. The Ct eluting from the sorbent until the feed termination ($Ct_{elution}$) is calculated by integrating under the elution curve using the mid-point rule:

$$Ct_{elution} = \sum_{t=0}^{t_s} \frac{C_n + C_{n-1}}{2}(t_{n-1} - t_n) \quad (2)$$

Where t_s is the time to saturation, C_n is the concentration eluting at time n, and C_{n-1} is the concentration at time n-1. W_E, the effective loading to saturation, is calculated by the following:

$$W_E = \frac{CT_{feed} - Ct_{Elution})F_{feed}}{M_{ads}MW} \quad (3)$$

Where M_{ads} is the mas of the adsorbent, MW is the molecular weight of the chemical in the feed (17 g mol^{-1} for NH_3), and F_{feed} is the feed flow rate.

Aging of Samples

Each metal-organic framework (MOF) mixed matrix membrane (MMM) was aged by placing the sample in an HD-205 Associated Environmental Systems environmental chamber set to 25° C and 90 % RH. Each sample was aged for 1, 3, 7, 14, and 28 days, then removed for further characterization.

Powder X-ray diffraction (PXRD)

Each MOF sample was analyzed using powder X-ray diffraction (PXRD). PXRD measurements were taken using a Rigaku Miniflex 600 X-ray powder diffractometer with a D/Tex detector. Samples were scanned at 40 kV and 15 mA, using Cu Ka radiation (λ = 1.54 Å), and a scan rate of 5° min^{-1} over a 2θ range of 3 to 50°. Double-sided tape on zero-background discs were used to affix the MMM and minimize background scattering. A background correction was performed in the Rigaku PDXL software (version 2.1.3.6).

Attenuated total reflectance Fourier-transform infrared spectroscopy (ATR-FTIR)

Attenuated total reflectance Fourier-transform infrared (ATR-FTIR) spectra of each MOF were taken using a Bruker Tensor 27 FTIR with a Bruker Platinum ATR accessory equipped with a single reflection diamond crystal. Sixteen scans were averaged over a range of 4000 to 400 cm^{-1} with a resolution of 4 cm^{-1}.

Scanning Electron Microscopy (SEM)

Scanning electron microscopy images were taken of each MMM after aging for 1, 3, 7, 14, and 28 days using a JEOL JCM-5700 Scanning Electron Microscope. Samples were supported on double-sided carbon tape and sputter coated with gold prior to analysis. The instrument was operated in high vacuum mode using an accelerating voltage of 15 kV at a nominal working distance of 10 mm.

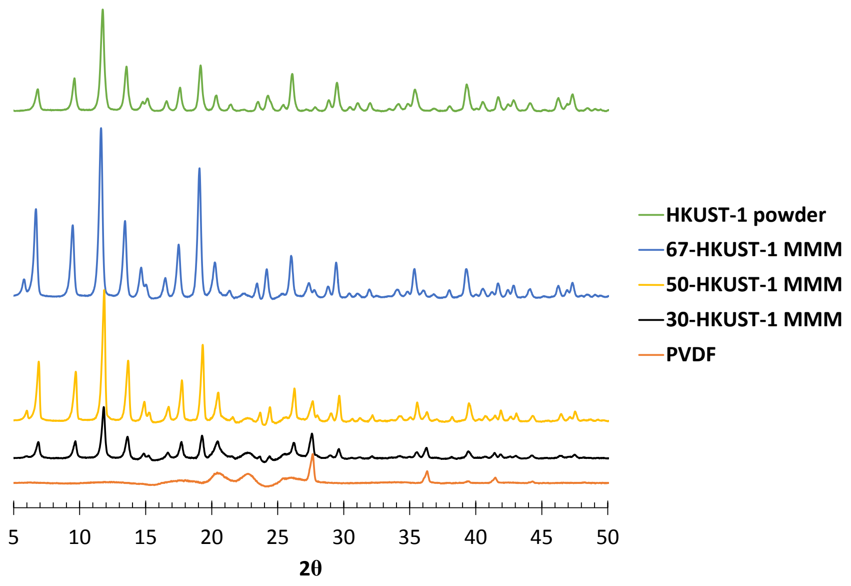

Figure S1. PXRD spectra of HKUST-1 MMMs compared to HKUST-1 powder and PVDF.

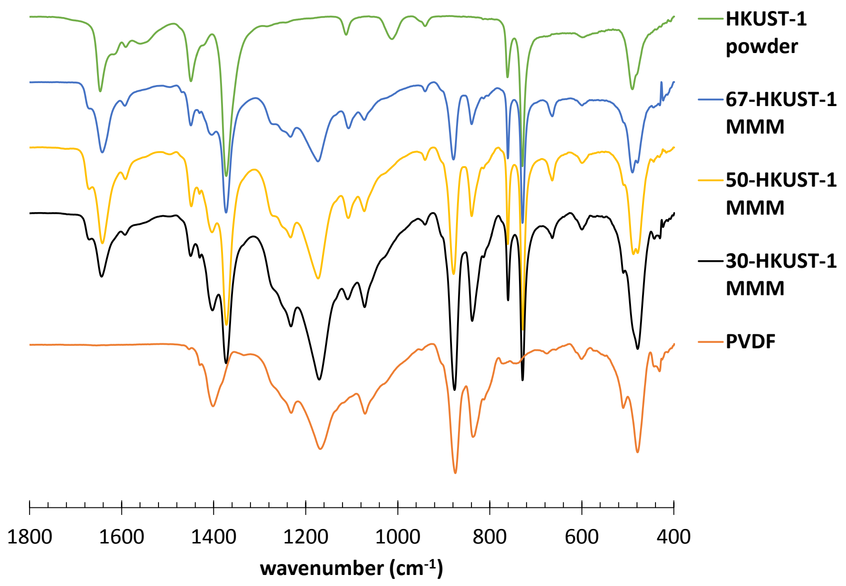

Figure S2. FTIR spectra of HKUST-1 MMMs compared to HKUST-1 powder and PVDF.

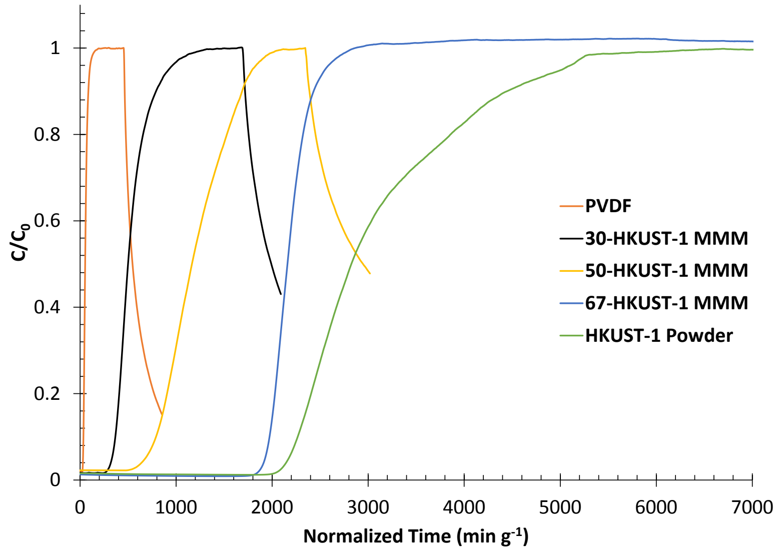

Figure S3. Microbreakthrough curves comparing PVDF polymer, HKUST-1 MMMs, and HKUST-1 powder.

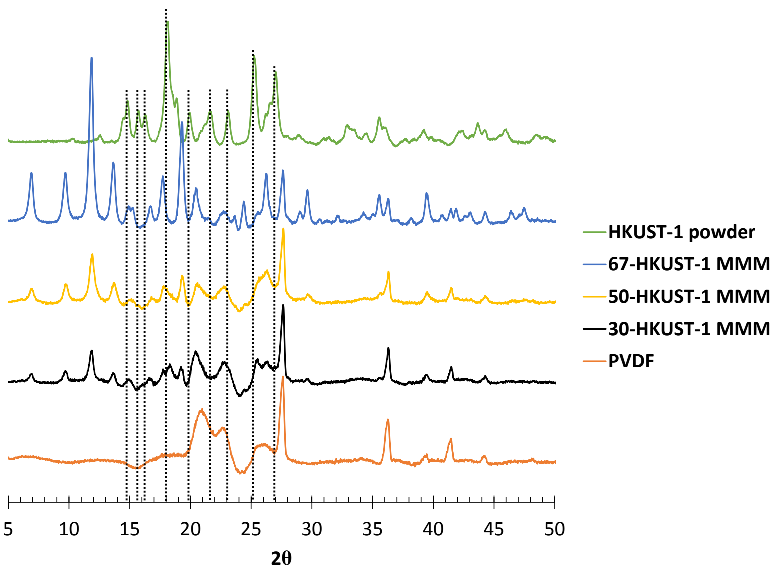

Figure S4. PXRD spectra of the HKUST-1 MMMs compared to HKUST-1 powder and PVDF after exposure to ammonia. The major reflections from the ammonia exposed HKUST-1 powder, are designate with lines at 2θ ≈ 14.8, 15.6, 16.2, 18.1, 19.9, 21.6, 22.1, 25.3, and 27.0°. There is no evidence of this change in the crystal structure in any of the ammonia exposed HKUST-1 MMMs.

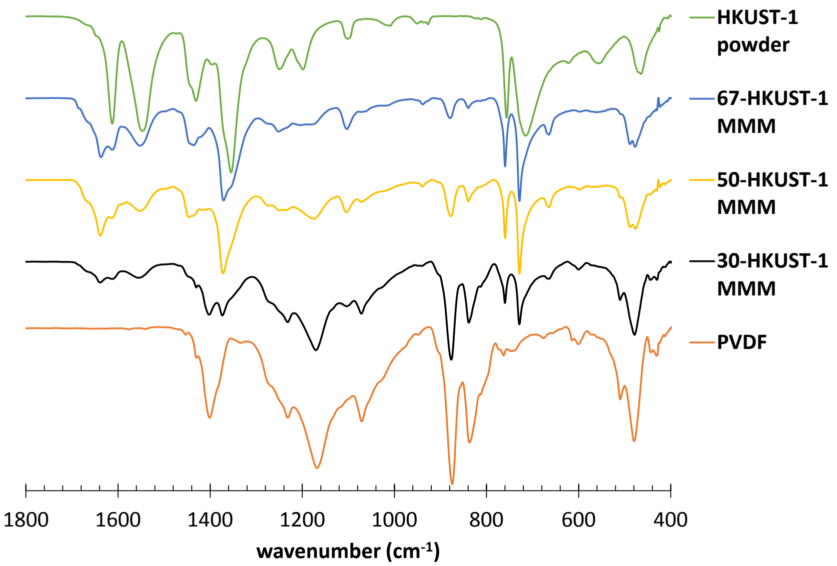

Figure S5. FTIR spectra of the HKUST-1 MMMs compared to HKUST-1 powder and PVDF after exposure to ammonia.

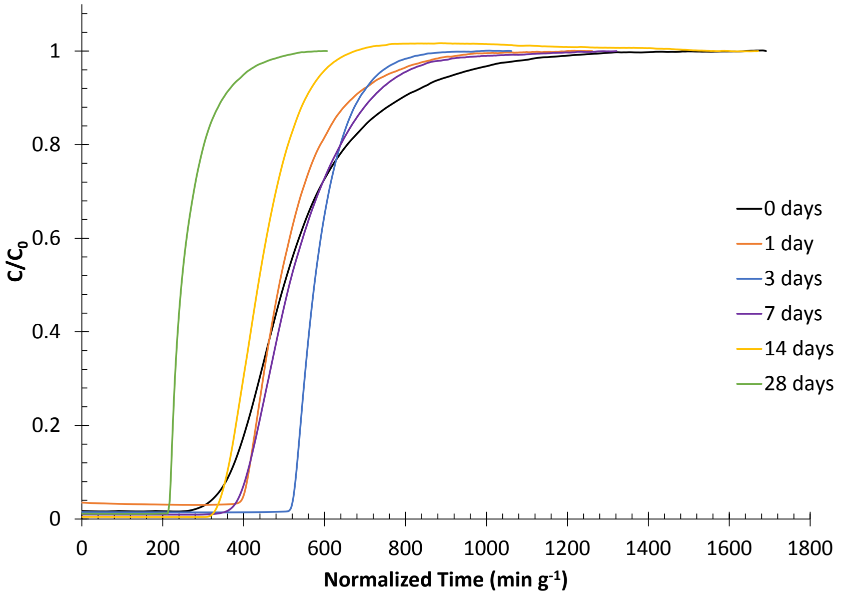

Figure S6. Ammonia breakthrough curved 30-HKUST-1 MMM aged for 0, 1, 3, 7, 14, and 28 days at 90% RH at 25 °C.

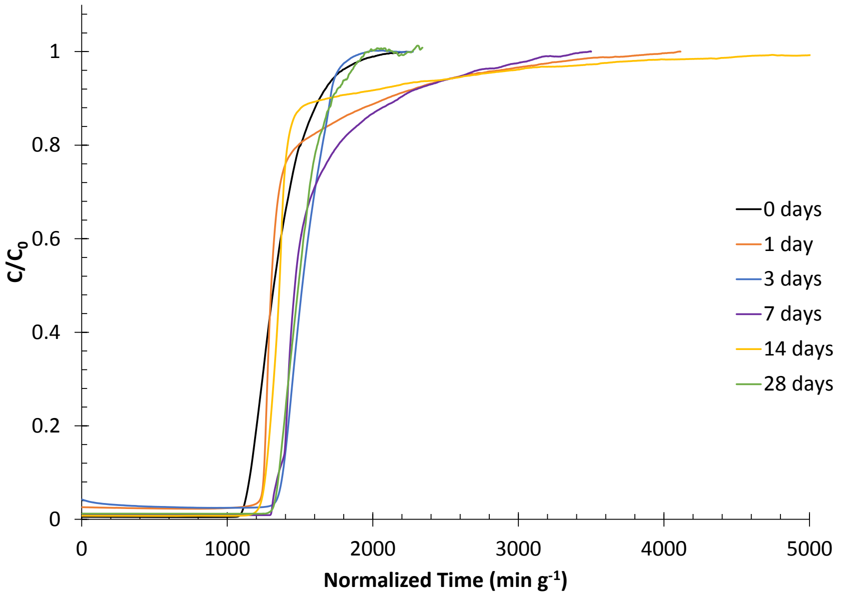

Figure S7. Ammonia breakthrough curved 50-HKUST-1 MMM aged for 0, 1, 3, 7, 14, and 28 days at 90% RH at 25 °C.

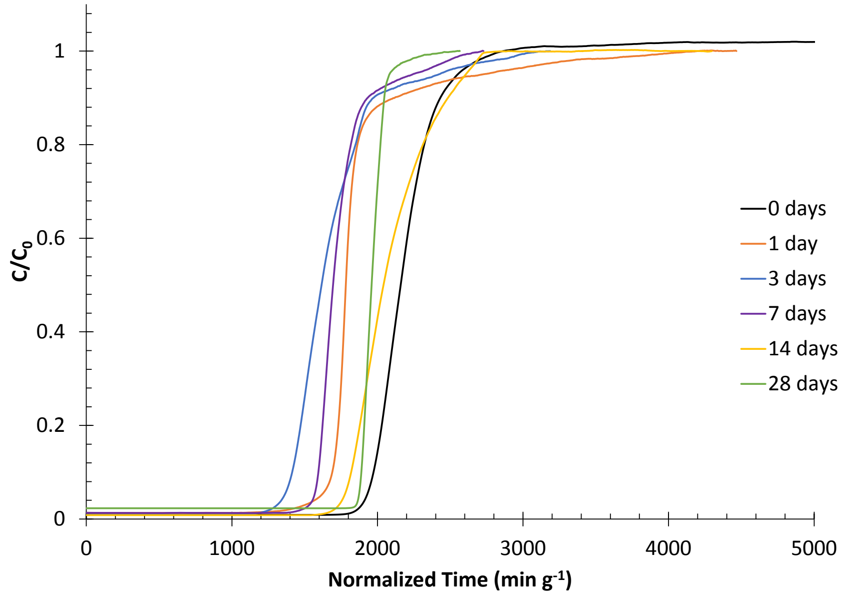

Figure S8. Ammonia breakthrough curved 67-HKUST-1 MMM aged for 0, 1, 3, 7, 14, and 28 days at 90% RH at 25 °C.

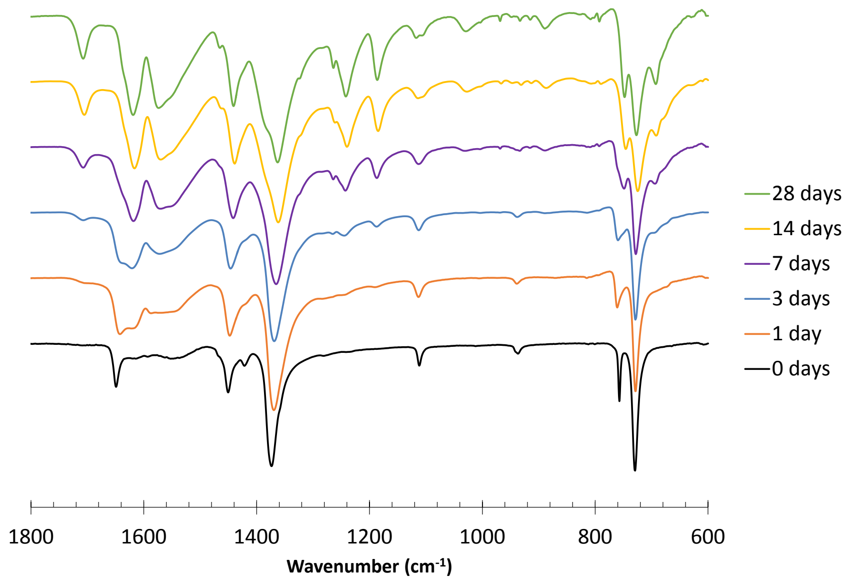

Figure S9. FTIR spectra of HKUST-1 before and after aging at 90% RH at 25 °C for 1, 3, 7, 14, and 28 days.

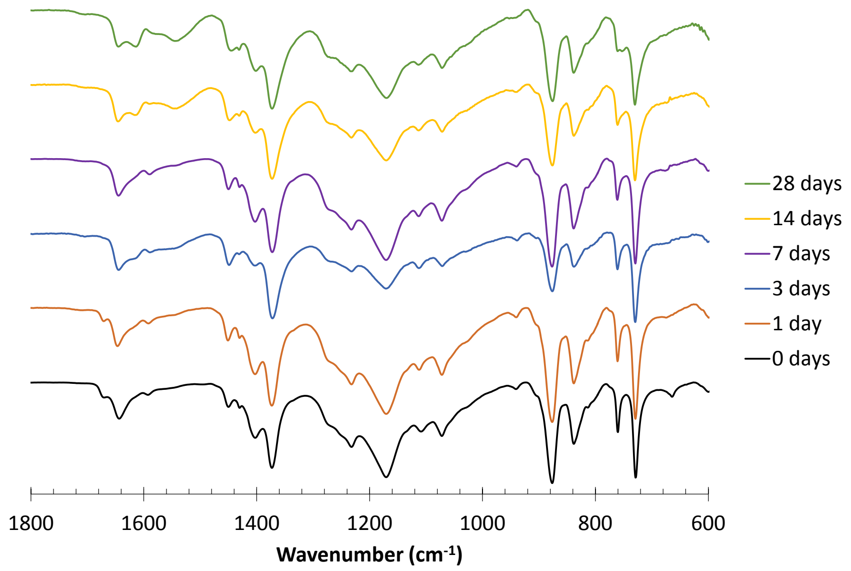

Figure S10. FTIR spectra of 30-HKUST-1 MMM before and after aging at 90% RH at 25 °C for 1, 3, 7, 14, and 28 days.

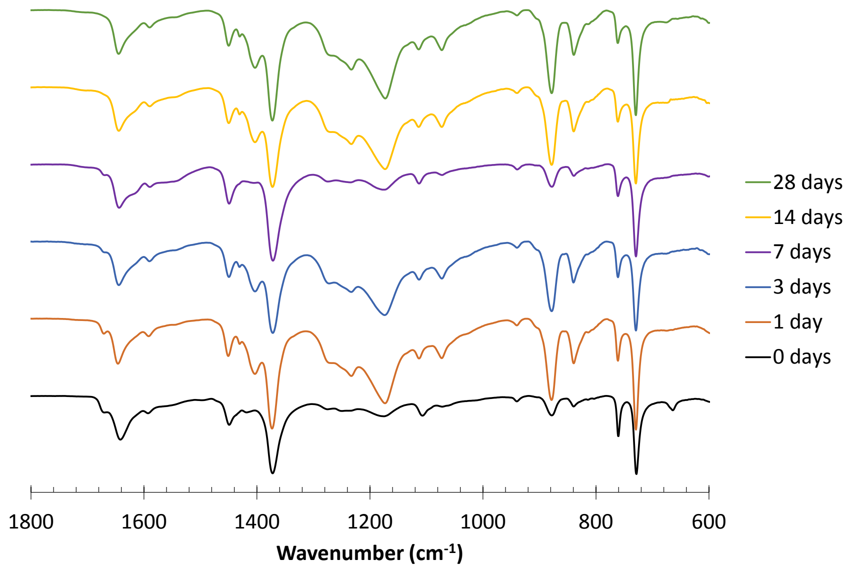

Figure S11. FTIR spectra of 50-HKUST-1 MMM before and after aging at 90% RH at 25 °C for 1, 3, 7, 14, and 28 days.

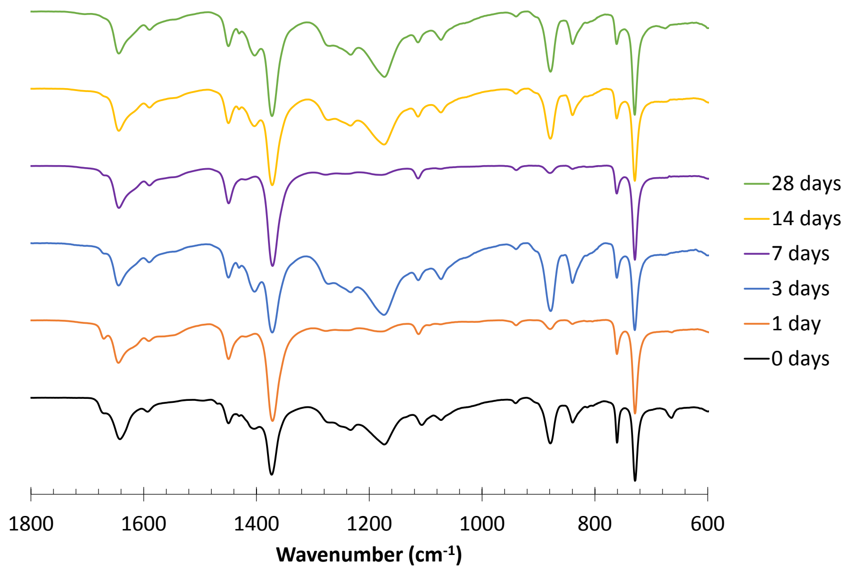

Figure S12. FTIR spectra of 67-HKUST-1 MMM before and after aging at 90% RH at 25 °C for 1, 3, 7, 14, and 28 days.

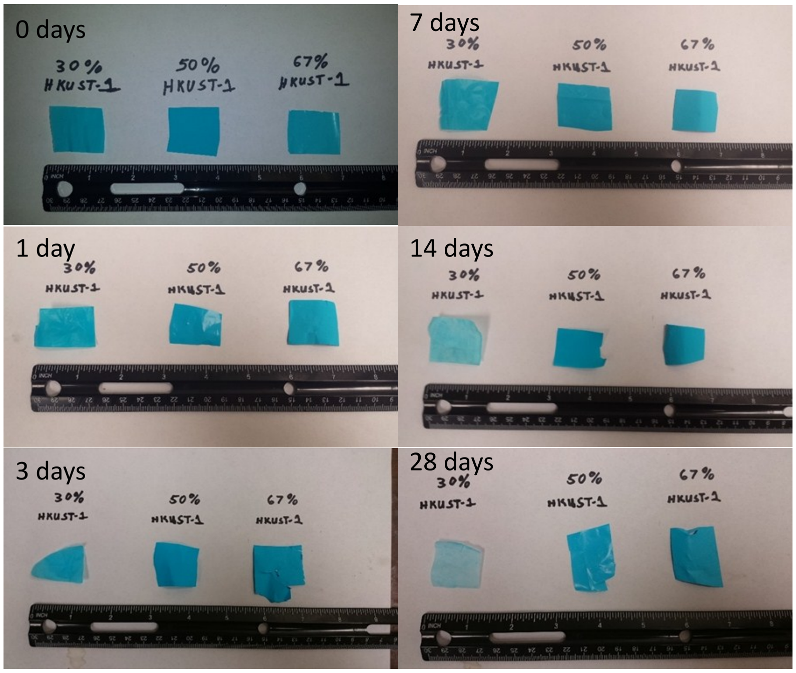

Figure S13. Photographs of each HKUST-1 MMM after aging for 0, 1, 3, 7, 14, and 28 days at 90% RH and 25 °C.

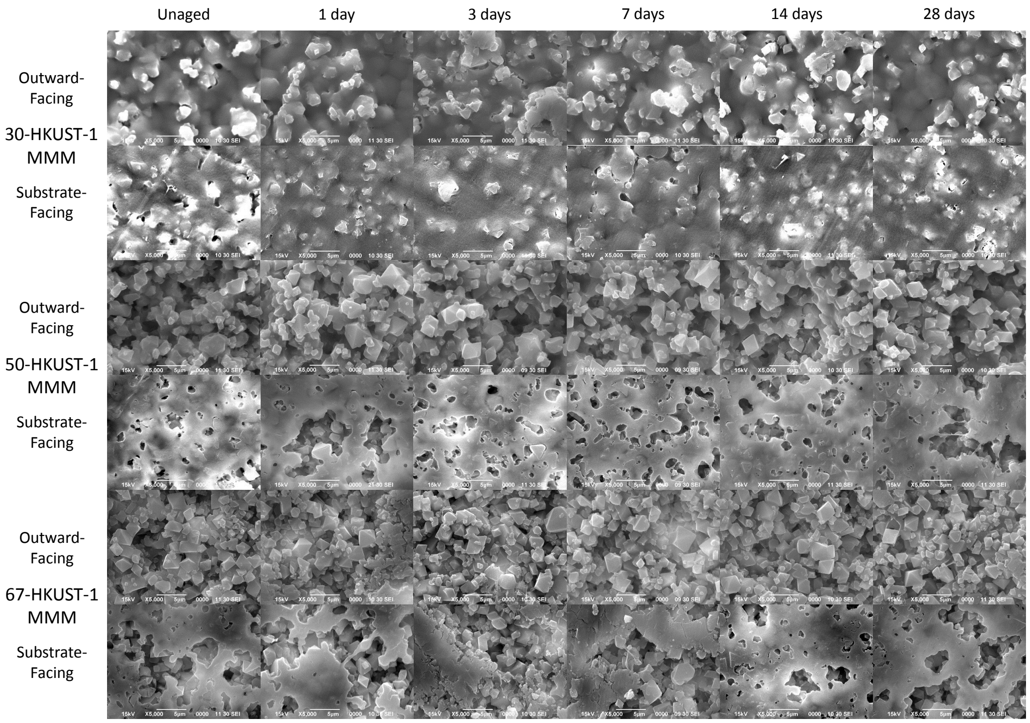

Figure S14. SEM images (X5000) of each HKUST-1 MMM, on the outward-facing (MOF dominant) side and substrate-facing (polymer dominant) side, after aging for 0, 1, 3, 7, 14, and 28 days, at 90% RH and 25 °C.

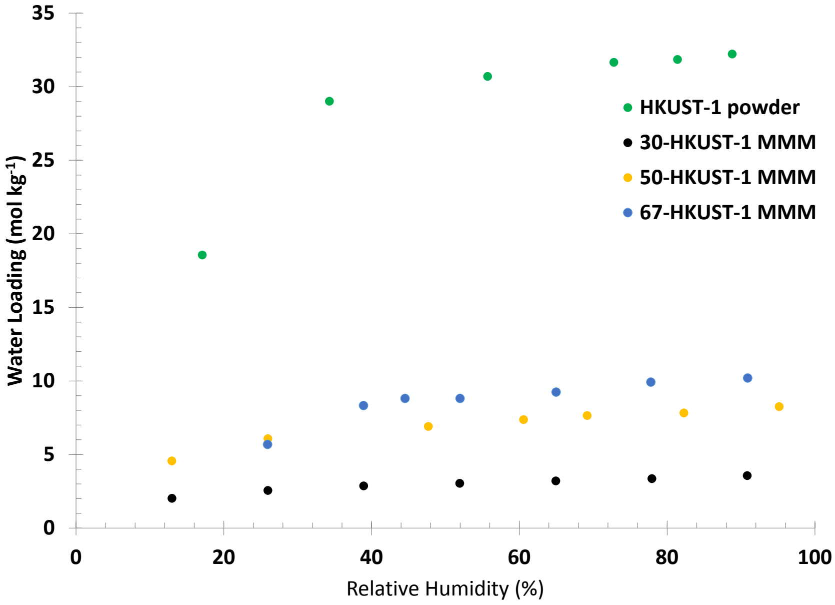

Figure S15. Water isotherms measured at 25 °C for HKUST-1 powder and each of the HKUST-1 MMMs.

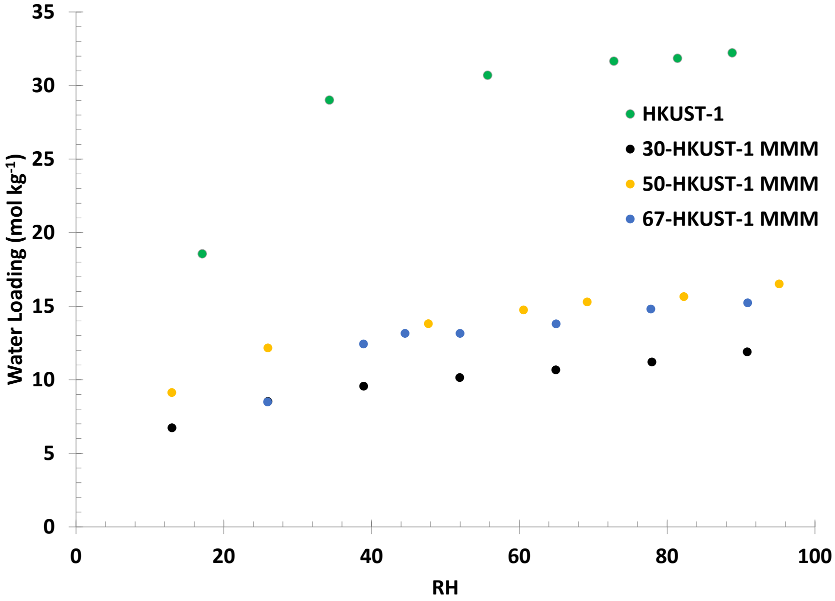

Figure S15. Water isotherms measured at 25 °C for HKUST-1 powder and each of the HKUST-1 MMMs, water loading is corrected for the amount of HKUST-1 present in the material.

(1) Zhuang, J.-L.; Ceglarek, D.; Pethuraj, S.; Terfort, A. *Adv. Funct. Mater.* **2011**, *21*, 1442.
(2) Denny, M. S.; Cohen, S. M. *Angewandte Chemie International Edition* **2015**, *54*, 9029.
(3) Glover, T. G.; Peterson, G. W.; Schindler, B. J.; Britt, D.; Yaghi, O. *Chem. Eng. Sci.* **2011**, *66*, 163.
(4) Decoste, J. B.; Peterson, G. W. *Journal of Visualized Experiments* **2013**, e51175.
